# Prevalence and behavioural risks for HIV and HCV infections in a population of drug users of Dakar, Senegal: the ANRS 12243 UDSEN study

**DOI:** 10.7448/IAS.18.1.19888

**Published:** 2015-05-22

**Authors:** Annie Leprêtre, Idrissa Ba, Karine Lacombe, Maryvonne Maynart, Abdalla Toufik, Ousseynou Ndiaye, Coumba Toure Kane, Joël Gozlan, Judicaël Tine, Ibrahim Ndoye, Gilles Raguin, Pierre-Marie Girard

**Affiliations:** 1 Institut Médecine Epidémiologie Appliquée, Université Xavier Bichat, Paris, France; 2 Hôpital Psychiatrique de Thiaroye, Dakar, Senegal; 3 Sorbonne-Universités, Paris, France; 4 Service de Maladies Infectieuses et Tropicales, Hôpitaux Universitaires de l'Est Parisien, Hôpital Saint-Antoine, Paris, France; 5 Inserm UMR-S1136, Institut Pierre-Louis de Santé Publique, Paris, France; 6 Centre Régional de Recherche et de Formation à la Prise en Charge Clinique (CRCF), Service des Maladies Infectieuses, Centre Hospitalier Universitaire de Fann, Dakar, Sénégal; 7 Laboratoire de Bacterio-virologie, Hôpital Le Dantec, Dakar, Senegal; 8 Laboratoire de Virologie, Hôpitaux Universitaires de l'Est Parisien, Hôpital Saint-Antoine, Paris, France; 9 Département Santé, Expertise France, Paris, France

**Keywords:** human immunodeficiency virus, hepatitis C virus, hepatitis B virus, drug use, Senegal, respondent-driven sampling

## Abstract

**Objectives:**

Data on the extent of drug use and associated HIV, hepatitis C and hepatitis B infection in West Africa are lacking. The objectives of ANRS12244 UDSEN study were to estimate the size of the heroin and/or cocaine drug user (DU) population living in the Dakar area (Senegal), and assess the prevalence and risk factors of HIV, hepatitis C virus (HCV) and hepatitis B virus (HBV), including behavioural determinants in this population, in order to set up an integrated prevention and treatment programme for DUs.

**Design and methods:**

A capture-recapture method was applied for population size estimation, whereas the respondent-driven sampling (RDS) method was used to recruit a sample of DUs living in the Dakar area and determine HIV, HBV and HCV prevalence. Behavioural data were gathered during face-to-face interviews, and blood samples were collected on dried blood spots for analysis in a central laboratory. Data analysis was performed using the RDS analysis tool, and risk factors were determined by logistic regression. Access to laboratory results was organized for the participants.

**Results:**

The size of the DU population in the Dakar area was estimated to reach 1324 (95% confidence interval (95% CI: 1281–1367)). Based on the 506 DUs included in the study, the HIV, HCV and HBV prevalence were 5.2% (95% CI: 3.8–6.3), 23.3% (95% CI: 21.2–25.2) and 7.9% (95% CI: 5.2–11.1), respectively. In people who inject drugs (PWID), prevalence levels increased to 9.4% for HIV and 38.9% for HCV (*p*=0.001 when compared to those who never injected). Women were more at risk of being HIV infected (prevalence: 13.04% versus 2.97% in males, *p*=0.001). Being PWID was a risk factor for HCV and HIV infection (odds ratio, OR: 2.7, 95% CI: 1.7–4.3, and OR: 4.3, 95% CI: 1.7–10.7, respectively), whereas older age and female sex were additional risk factors for HIV infection (10% increase per year of age, *p*=0.03 and OR: 4.9, 95% CI: 1.6–156, respectively). No specific determinant was associated with the risk of HBV infection.

**Conclusions:**

High HIV and HCV prevalence were estimated in this population of DUs (including non-injectors) living in the Dakar area, Senegal, whereas HBV prevalence was close to that of the global Senegalese population, reflecting a risk of infection independent of drug use. Women seem to be highly vulnerable and deserve targeted interventions for decreasing exposure to HIV, while behavioural risk factors for HIV and HCV include the use of unsafe injections, reflecting the urgent need for developing harm reduction interventions and access to opioid substitution therapy services.

## Introduction

Until recently, illicit drug use was not considered an important route of HIV transmission in sub-Saharan Africa. Consequently, coverage of HIV prevention, treatment and care services to drug users (DUs) populations continued to be very low, with the lowest rates reported in sub-Saharan Africa [1]. However, recent data clearly demonstrate that the number of countries in which people use illegal drugs (including by injection) is growing, fuelling the HIV epidemic. In 2010, during the XVIII International AIDS Conference, the Vienna declaration called on governments and international organizations to urgently reorient drug policies and implement science-based public health approaches to address the harm stemming from drug injecting (www.sciencedaily.com/releases/2010/06/100628101446.html, last accessed 10 March 2015). The United Nations Office on Drugs and Crime has issued warnings on the increasing trafficking and use of drugs in sub-Saharan Africa, including West Africa [2]. A systematic review in 2008 on injecting drug use and HIV did not report any data from francophone African countries, whereas scarce data were collected from Kenya, Nigeria and South Africa [1], where the HIV prevalence among people who inject drugs (PWID) was, respectively, estimated at 42.9, 5.5, and 12.4% and more recently at 34.8% in Tanzania [3]. In the plea “Time to act” [4], authors highlighted evidence from several sub-Saharan African countries where the use of injecting drugs was rapidly increasing and was associated with a spread of HIV infection. A cross-sectional study conducted in 2010 in six states in Nigeria provides the only data available for PWID in West Africa and reported the high heterogeneity in the prevalence across states, from 0.3 to 9.3%, with a prevalence higher than 5% in three of the six states [5]. Moreover, studies conducted in sub-Saharan Africa have demonstrated that there is a strong association between the risk of HIV acquisition and alcohol use [6] and that consumption of stimulant drugs such as cocaine or crack induces high-risk sexual behaviours [7,8].

Despite the threat of a higher prevalence and transmissibility of viral hepatitis among PWID, detailed data on hepatitis C virus (HCV) are rare in sub-Saharan Africa, as noted in a systematic review in 2011 [9]. Prevalence of HCV antibodies in PWID was available in only 4 of the 17 countries where injecting drug use has been identified. As expected, HCV prevalence was much higher than HIV within a range of 97.3% in Mauritius, 51.4% in Kenya, 40.1% in Ghana and 22.2% in Tanzania. Last, non-injecting drug use is also recognized as an emerging risk factor for HCV acquisition [10] with a prevalence ranging from 2.3 to 35.3%.

In Senegal, the HIV epidemic is concentrated with a low prevalence in the general population (0.7% with a 1.6 female/male ratio) [11] and a high prevalence among key populations most at risk of HIV exposure, such as female sex workers (18.5%) and men who have sex with men (21.8%) [12]. However, no data on HIV prevalence among DUs are available despite the fact that injecting drug use was reported in specific areas of the Dakar region [13]. Data on HCV prevalence are even scarcer. A recent review reported a 3% rate in the general population [14], whereas a cross-sectional study of a blood donor population found a 0.49% rate of HCV antibodies [15]. A local survey conducted in 2008 to 2009 has shown that smoking is the main route of consumption of heroin, cocaine and crack [16]. Furthermore, those who do not inject are often connected to PWID. This might constitute a reservoir for HCV and HIV transmission through the community, inasmuch as crack use has been proved to be a risk factor for HIV and HCV in Europe [17].

Therefore, we designed a survey to estimate the size of the heroin and/or cocaine DU population living in the Dakar area (whatever the patterns of use), to assess HIV and HCV prevalence in this population and to analyze related behavioural risk factors in order to set up an integrated prevention and treatment programme for DUs.

## Patients and methods

### Study design and participants

The Dakar area is the most populated region of Senegal, accounting for 23% of the total population (*n*=12,526,488) and 75% of the urban population [11]. The legal and social context of drug use is repressive, and there is no access to opioid substitution treatment and specialized care.

The study, conducted between April and July 2011, had two components: a capture-recapture component that estimated the size of the Dakar region's DU population, and a cross-sectional survey based on the respondent-driven sampling (RDS) methodology to estimate HIV and HCV prevalence within this population. DUs were defined as having used heroin and/or cocaine or injected any psychoactive substance during the last three months. The capture-recapture method consists of a random distribution of a numbered token within the target population during the week preceding the cross-sectional survey and its recapture within the population included in the survey. RDS is a long-chain referral methodology that has been developed to provide access to difficult-to-reach populations [18]. Recruitment starts with the so-called “seeds” known to be representative of the DU population's diversity. Seeds are instructed to give a recruitment coupon to a maximum of three other peers from their own social network and, in turn, they receive double incentives, one for their participation and one for succeeding in recruiting peers. Limiting the number of peers who could be recruited by each participant avoids biases associated with the snowballing sampling method, which has the drawback of increasing the risk of not reaching hidden individuals. Recruitment proceeds in successive waves of recruited peers. Using a mathematical system for weighting the sample to compensate for its not having been drawn as a simple random sample, a final population sample is obtained and used to measure study outcomes.

Inclusion criteria were as follows: being a heroin and/or cocaine user, having injected any psychoactive substance during the last three months, being over 18 years of age (or 15 in cases of proven emancipation), living for at least three months in the Dakar region, and giving informed consent to participate in the study. In order to minimize the risk of including the same participant twice, three basic biometric measures (height, size of wrist and distance from elbow to end of third finger) were reported on a computerized database [19]. The initial sample size was fixed at 266 individuals (design effect of two with unilateral test, initial indicator P1=0.50 and P2=0.65, with an α risk of 5% and an 80% power).

### Study procedures

The study was preceded by a feasibility survey (December 2008 to January 2009), aimed at identifying institutional key partners (Ministry of Health, CILD, CNLS, PWVIH, NGO, and care and treatment centres), identifying and briefly describing the target population, identifying the study centre and assessing the needs for survey staff training. An outreach team including former DUs acting as peers, social workers and volunteers conducted field work for six months to increase trust between DU communities and interviewers and to have a better understanding of the DU network in the Dakar area.

The capture-recapture study preceded the RDS survey by one week (11 to 17 April 2011). Tokens were randomly distributed as pre-numbered cardboards in the different spots where DUs usually gathered, to all DUs matching the inclusion criteria of the RDS study, and then recaptured during the RDS survey. Participants of the study were not informed on the reason why tokens were distributed, on the award when bringing the token back or on the implementation of the RDS study. This ensured the independence of both samples. The total number of tokens distributed in the community has been precisely noted (capture) in order to estimate the number of tokens collected from patients presenting to the study research base during the RDS study (recapture).

During the RDS survey phase (19 April to 15 July 2011), two to three seeds per area covered by the study were initially selected for a total of eight seeds. Of all seeds recruited per area, one had to be a woman, one under 25 years of age, one a PWID, one a heroin user and one a crack user (those characteristics could be combined). Each recruited seed received a maximum of three coupons to be distributed to peers. The study research base was located on the Fann University Hospital premises. Each eligible individual filled out a face-to-face behavioural questionnaire and had a blood sample collected on a dried blood spot (DBS). The questionnaire was administered in the most common language (Wolof) using a back translation from French, following the method described here: [www.who.int/substance_abuse/research_tools/translation/en/]. The sociobehavioural questionnaire contained items regarding sociodemographical characteristics, present and past drug use practices, knowledge of HIV and HCV transmission modes, access to care and testing, sexual risk behaviours and previous jail experience. Compensation for participation (4000 FCFA) and transport (3000 FCFA) was set at a total amount of 7000 FCFA (11 €) and given when visiting the study centre, whereas the facilitators who recruited DUs in the survey received 4000 FCFA (6 €), provided that the DU inclusion was effective.

During the recruitment, DBS were prepared from finger pricks. For each patient, five spots were placed on a five-spot blotter 903^®^ (Whatman GmbH, Dassel, Germany) and dried for at least three hours at room temperature. They were then centralized in the virology lab of the study centre (CRCF) in Dakar. Most of the DBS were kept at 4°C for two weeks before being frozen. HIV testing was done according to the national algorithm using the Determine^®^ HIV-1/2 Ag/Combo kit (Alere Medical Co., Matsuhidai, Matsudo-shi, Chiba, Japan) and confirmed with ImmunoComb^®^ II HIV1 & 2 BiSpot kit (Orgenics, Yavne, Israel). Antibody elution from the DBS was performed in 200 µl of the PBS buffer, and 50 µl was used for each technique according to the manufacturer's instructions. HCV and HBV serology were performed, respectively, with the VEDALAB-HCV-CHECK-1 kit and HEP-CHECK-1 kit (VEDA LAB, Alencon, France). For each technique, 100 and 20 µl were used for HBV and HCV, respectively, according to the manufacturer's instructions.

### Outcomes

The primary outcomes were the estimation of the size of the DU population within the Dakar area and the prevalence of HIV, HCV and HBV with its 95% confidence interval (95% CI). Secondary outcomes included the prevalence of HIV, HCV and HBV infection in the study population based on serologies performed from DBS, and the assessment of the relative risk of HIV, HBV and HCV infections based on the information obtained through the sociobehavioural questionnaire.

### Statistical analysis

RDSAT version 5.6 was used to estimate the prevalence of HIV, HCV and HBV infections based on the sample population and allowed adjustments on parameters such as homophily and difference in network size, known to cause possible biased estimates.

The capture-recapture method was applied to the estimation of the total size of the DU population in Dakar. The estimation was based on the equation: *N*=(*n*1×*n*2)/*m*, where *n*1 is the number of DUs who got a token (capture), *n*2 is the number of DUs included in the RDS study (recapture) and *m* is the number of participants common to both groups (DUs with a token).

Descriptive statistics were applied to the characteristics of the survey population. Categorical variables were reported with their percentages and compared using the chi-squared test (and Fisher's test for small numbers). Continuous variables were expressed by their means (standard deviation) or medians (interquartile range), and comparisons were with the Student and Wilcoxon ranked tests according to the variable distribution. A simple logistic regression was applied to select variables associated with the outcome, and all variables with *p<*0.1 were entered in a multiple logistic regression model for each outcome. Statistical analysis was performed using STATA (v11.2; College Station, TX, USA), and significance was determined as *p<*0.05.

### Ethical considerations

All participants provided informed oral and written consent to participate in the study. After answering the questionnaire, individuals had a pretest counselling session about HIV and HCV prevention. Within one week, the blood test results were confidentially delivered in the Outpatient Treatment Centre (Centre de Traitement Ambulatoire) near the research centre. Participants also received post-test counselling and all HIV- and/or HCV- or HBV-positive patients were offered an appointment free-of-charge with an infectious diseases specialist in the same hospital. Counselling and blood tests were also offered to non-included individuals. Anonymity was ensured throughout the process by the attribution of a unique number with no connection to identity, unless the participant who turned out to be HIV, HBV or HCV infected, accepted the appointment with the infectious diseases specialist. Finally, all study partners discussed the appropriate amount for incentives distributed to recruiters and defined it according to common practices and rates for Senegalese clinical research. The study protocol was reviewed and approved by the National Ethical Committee for Research in Health.

## Results

### Population size and HIV, HBV and HCV prevalence

The estimation of the population size has been performed based on 416 distributed tokens. The estimated DU population in Dakar vicinities was an average of 1324 (95% CI: 1281–1367).

In total, 625 DUs were recruited from the eight initial seeds, among which 506 have been included in the survey. Among the 119 non-included individuals, 81 could not adequately prove that they were active DUs of heroin and/or cocaine/crack (most of them were using cannabis), 26 had not used drugs in the past three months and 12 were excluded for other reasons (fear of blood tests, no time for the questionnaire, etc.). In total, 610 had a serological test performed (15 refused to be tested). As of 15 November 2011, 226 participants (37%) came to get their results. Compared to those who did not come back, they were more often males (39.5% versus 17.2%, *p<*0.001), older (43.8 versus 34.1 years of age, *p<*0.001), more susceptible to be included in the study (41.5% versus 13.4%, *p*<0.001) and less likely to have injected drugs within the past six months (30.7% versus 43.8%, *p=*0.02).

The prevalence of HIV, HBV and HCV in the DU population living in the Dakar area were estimated to be 5.2% (95% CI: 0.038–0.063), 7.9% (95%CI: 5.2–11.1) and 23.3% (95% CI: 0.212–0.252), respectively.

In the sample (*n*=506), the total number of HIV, HBV and HCV infection was 22 (4.3%), 45 (8.9%) and 120 (23.8%), respectively. The HIV prevalence was significantly different between DUs having injected drugs at least once in their life and those who never injected: 9.4% versus 2.5% (*p*=0.001), women and men: 13% versus 3% *(p*=0.001), even for those having injected drugs at least once: 21.1% versus 7.5% (*p*=0.0001). The HCV prevalence was significantly different between DUs who injected drugs at least once in their lives and those who never did: 38.85% versus 18% (*p*=0.0001). Sex did not influence the prevalence of HCV (32.0% in females versus 22.9%, *p=*0.2) or HBV (4.3% in females versus 9.6%, *p*=0.2). Age was marginally positively associated with HIV prevalence (*p*=0.007), but not with HCV or HBV prevalence.

Among the 22 HIV-positive patients from the study sample, 13 (59.1%) and 2 (9%) were also HCV or HBs-Ag co-infected, respectively. Conversely, among the 120 HCV-positive and 45 HBs-Ag positive patients from the study sample, the prevalence of HIV-HCV or HIV-HBV co-infections was 10.8% and 4.4%, respectively.

### Characteristics of the survey population

The survey population was predominantly male (*n*=437, 86.4%) with a mean age of 42.1 years (SD 10.4). Most DUs (*n*=356, 70.4%) were single, divorced or widowed. The education level was relatively low, in accordance with the reported rates in the general population [11], 51.2% (*n*=259) had none or went only to primary or Koranic school, but 18.9% had attended high school and/or university. The majority (*n*=296, 58.6%) lived with their parents and only four had no stable housing during the previous three months. A small majority (*n=*300, 59.3%) had a paid occupation. A history of incarceration was reported by 61.9% of participants, and 29.2% of them acknowledged having consumed drugs within the jail premises. Among all participants, 27.7% (*n*=140) reported having injected drugs at least once in their lives, and 38.9% (*n*=197) had already received care for addiction. Only 29.4% had been tested for HIV (none reporting a positive result), 4.2% for HBV and 3.9% for HCV (one reported a positive result).

In the month prior to survey, the use of drugs was as follows: heroin=91.5%, cannabis=64%, crack=49.4%, alcohol=49%, benzodiazepines=29.8% and cocaine=13.6%. Consumption of more than three drugs was noted in 291 (57.5%) DUs. The concomitant use of pain killers such as paracetamol was frequent (26.9%), and 27 patients reported occasional opiate substitution consumption (two methadone and five buprenorphin), although these drugs had no legal existence in 2011 in Senegal.

Mean age at heroin, crack and cocaine initiation was 27.9 years (SD: 8.2), 28.9 years (SD: 8.5) and 29.3 years (SD: 8.3), respectively. However, initiation of heroin and crack use occurred before the age of 22 and 23, respectively, in 25% of the participants. Cannabis and alcohol consumption began earlier, at a mean age of 20.5 years (SD: 7.0) and 21.4 years (SD: 7.4), respectively, whereas benzodiazepines consumption began later at a mean age of 31.5 years (SD: 11.0). For those who had ever injected once, the first injection occurred at a mean age of 30.8 years (SD: 8.2).

Among the participants who used heroin, 367 (72.5%) would take it every day, whereas crack was consumed once a week or less by 176 participants (70.4% of crack users), and cocaine once a week or less by 43 participants (62.3% of cocaine users). Cannabis was also consumed daily by 202 participants (62.4%), as was alcohol and benzodiazepines (33.1% and 29.8% of daily consumption, respectively).

The most frequent route of heroin absorption was smoking (68.0%), then snorting (52.1%) and injection (14.0%). Cocaine was mainly smoked (71.0%), followed by snorting (43.5%) and injection (15.9%). Crack and cannabis were smoked (100 and 97%, respectively). Injections never occurred for pills such as benzodiazepines. For participants who injected, the date of the last injection was within the month preceding the study (70%), and 12.9% shared needles with other injectors, whereas more than a quarter of them shared other injection materials (water used for drug dilution: 27.1%; water used to wash equipment: 21.4%; spoon 34.3% and filter: 27.1%). Personal syringes were re-used by 60% of injectors, after cleaning in 72% of cases, mostly with water (60%). For 33.9%, the last injection occurred outside the home (reflecting the precarious situation of some PWID). In the 86% of participants who smoked drugs within the past month (*n*=435), 60.2% have shared pipes or other oral devices, and in the 49% of participants who snorted (*n*=251), 27.1% have shared straws or other intranasal devices.

Regarding sexual intercourse, 80.4% of participants reported being sexually active within the last 12 months, of which 45.7% reported sex with more than one partner, and the majority (65%) rarely or never used condoms. One-time paid sexual relationships were reported by 26.8% of participants.

### Special focus on women

Women included in the study (*n*=69) were younger than men (36.1 versus 43.0 years, *p*=0.0001). A higher proportion had a precarious family situation (divorced, single, widowed or separated) (83% versus 68% for men, *p*=0.01) and no or a low level of education (71% versus 48% for men, *p<*0.0001). The two drugs most frequently used were heroin and crack (79% and 46% of all women, respectively). Women started injecting heroin at a younger age than men (25.6 versus 28.1 years, *p=*0.03). More women than men had had sex during the last 12 months (88.4% versus 79.0%, *p*=0.04), and a higher proportion of sexual relationships were associated with prostitution (62.3% versus 20.6% in men, *p*<0.0001).

### Knowledge and beliefs regarding use of drugs and risk of HIV or HCV infection

Table 1 reports the results of the knowledge and beliefs questionnaire. The majority of participants were aware of the main risk factors for HIV and HCV transmission (except for straws and crack pipes, which were mainly thought not to be HIV-transmission routes). Of note, 45% of participants believed mosquitos could transmit HIV.

**Table 1:** Knowledge and beliefs regarding HIV and HCV infections

	Number of participants answering yes to questions regarding HIV (*n*, %)	Number of participants answering yes to questions regarding HCV (*n*, %)
Transmission when using same syringe	487 (96.3)	395 (78.1)
Transmission when using same water used for diluting powder	390 (77.1)	364 (71.9)
Transmission when using same straw	218 (43.1)	297 (58.7)
Transmission when using same spoon	320 (63.2)	313 (62)
Transmission when using same water used for washing injecting equipment	418 (82.6)	382 (75.5)
Transmission when using same crack pipe	225 (44.5)	322 (63.6)
Transmission when using same cotton	385 (76.1)	364 (71.9)
Transmission through food	78 (15.4)	–
Transmission by mosquitoes	228 (45.1)	–
Transmission by person apparently in good state of health	451 (89.1)	–
Reduction in the risk of transmission with regular sexual partner	363 (71.7)	–
Reduction in the risk of transmission with use of condoms	447 (88.3)	–

### Risk factors for HIV, HBV and HCV infection

Regarding HIV infection (Table 2), being of female sex multiplied the risk of being infected by almost fivefold (odds ratio, OR=4.9, 95% CI: 1.6–15.6). Having injected drugs at least once in their lives was another significant determinant of HIV infection (OR=4.3, 95% CI: 1.7–10.7), and risk also increased with age (+10% per year of age). For HCV (Table 3), having injected drugs at least once in their lives was significantly associated with infection (OR=2.7, 95% CI: 1.7–4.3), as well as being single/divorced or widowed (OR=1.81, 95% CI: 1.08–3.04) and having maintained a longer duration of relationship with the study recruiter (OR=1.001, 95% CI: 1.0–1.002). No specific risk factors for HBV infection has been individualized in the study population.

**Table 2:** Risk factors for HIV infection

	HIV status						
						
Variables^a^	HIV+ (*n*=22)	HIV− (*n=*484)	*p*	Crude OR (95%CI)	*p*	Adjusted OR (95%CI)	*p*
Sex (*n*, %)							
Male	13 (59.1)	424 (87.6)		1		1	
Female	9 (40.9)	60 (12.4)	0.001	4.9 (2.01–11.9)	0.0001	4.9 (1.6–15.6)	0.007
Paid sexual relationships at least once (*n*, %)							
No	12 (54.5)	361 (74.6)		1		1	
Yes	10 (45.5)	123 (25.4)	0.09	2.4 (1.03–5.8)	0.04	1.5 (0.5–4.6)	0.4
Age, mean (SD), OR per year	46.0 (9.3)	41.9 (10.4)	0.07	1.04 (1.00–1.1)	0.08	1.1 (1.004–1.1)	0.03
History of injection at least once (*n*, %)							
No	9 (40.9)	357 (73.8)		1		1	
Yes	13 (59.1)	127 (26.2)	0.002	4.1 (1.7–9.7)	0.002	2.9 (1.06–7.9)	0.04
Use of heroin by injection (*n*, %)							
No	14 (63.6)	427 (88.2)		1			
Yes	8 (36.4)	57 (11.8)	0.004	4.3 (1.7–10.7)	0.002	–^b^	–

a Only variables associated with outcome at *p*<0.1 in univariate analysis are reported in the table

b because of a strong collinearity between the variables “injector status” and “use of heroin by injection,” only the first has been kept in the multivariable model.

**Table 3:** Risk factors for HCV infection

	HCV status						
						
Variables^a^	HCV+ (*n*=120)	HCV− (*n*=386)	*p*	Crude OR (95% CI)	*p*	Adjusted OR (95% CI)	*p*
Single, divorced, widowed (*n*, %)							
No	26 (22.7)	124 (32.1)		1		1	
Yes	94 (78.3)	262 (67.9)	0.03	1.7 (1.05–2.8)	0.03	1.81 (1.1–3.04)	0.02
Paid sexual relationships within past 12 months (*n*, %)							
No	81 (67.5)	292 (75.6)		1		1	
Yes	39 (32.5)	94 (24.4)	0.09	1.49 (0.95–2.3)	0.08	1.2 (0.9–1.9)	0.4
Care sought for heroin use (*n*, %)							
No	72 (60.4)	267 (69.2)		1		1	
Yes	48 (39.6)	119 (30.8)	0.08	1.6 (0.98–2.3)	0.06	1.2 (0.7–1.9)	0.5
Duration of relationship with study recruiter, days (mean, SD), OR per day				1.001 (1.0002–1.003)	0.02	1.001 (1.0–1.002)	0.05
History of injection at least once (*n*, %)							
No	65 (54.2)	301 (77.8)		1		1	
Yes	55 (45.8)	85 (22.2)	0.0001	3.0 (1.9–4.6)	0.0001	2.7 (1.7–4.3)	0.0001
Use of heroin by injection (*n*, %)							
No	89 (74.2)	352 (91.2)		1			
Yes	31 (25.8)	34 (8.8)	0.0001	3.6 (2.1–6.2)	0.0001	–^b^	–
Injection of drugs during trip abroad (*n*, %)							
No	104 (86.7)	362 (93.8)		1			
Yes	16 (13.3)	24 (6.2)	0.03	1.6 (0.8–3.3)	0.2	–	–

a Only variables associated with outcome at *p<*0.1 in univariate analysis are reported in the table

b because of a strong collinearity between the variables “injector status” and “use of heroin by injection,” only the first has been kept in the multivariable.

## Discussion

This study is the first of its kind conducted in West Africa and addressing the issue of drug use (heroin and/or cocaine/crack) in Dakar, Senegal, within a population that remains hidden and marginalized due to a repressive social and legal context. Based on the field team's previous work, the survey was well accepted by DUs, and there was good productivity from the selected “seeds” as well as fast recruitment by the peers. DUs expressed no problems regarding safety or breach of confidentiality, due to the study team's extensive training in research and confidentiality management. However, a fairly low number of participants came back to get their HIV, HBV and HCV test results. This emphasizes the lack of an accessible prevention and treatment programme of drug use and chronic hepatitis in West Africa, as well as the repressive legal and social context that prevents the participants from coming and getting their results. However, those who came back were males, older and more often non-injecting DUs, which underlines the excessive vulnerability of younger DUs, females and injectors who probably need to be reached by more specific programmes designed for difficult-to-reach populations.

Our study confirmed the existence of heroin and cocaine users in Senegal, estimated at 1300 in the Dakar area, numbering an average 3.8 million [20]. Heroin is the most frequently used opiate product through smoking, although 27.7% of users reported having injected at least once in their life. Heroin is also the most common injected drug, as in other sub-Saharan African countries [21]. Frequent polydrug use of psychotropic substances was also noted. We found surprisingly high consumption of alcohol (49% of DUs, 16.3% reporting a daily consumption), which began before heroin use, although Senegal is categorized as a low alcohol-consuming country [6]. The use of benzodiazepines that were never injected but used as painkillers began three years after the initiation of heroin and demonstrates the need for decreasing symptoms of opioid withdrawal syndrome. The majority of DUs were males and started to use heroin and cocaine at a later age than in Europe (27.9, 28.9 and 29.3 years of age for heroin, crack and cocaine, respectively, compared to a mean 22 years of age in Europe [22]). This is in line with data from Lagos and Oyo, Nigeria [5], where mean age at first injection was older than 30 years. However, in Tanzania, the mean age of initiation of heroin use was close to that observed in Europe, 20.4 and 25 years for men, and 19.5 and 22.8 years for women, respectively [23]. The reason of the use of injections at late age in Senegal is presently not clear. One might speculate that despite precarious living conditions, a very small fraction of the participants were homeless (4/506), most of them living with their families. The strength of family structure may prevent youngsters from turning to heavy drug use. Indeed, the DUs included in the study mostly lived in precarious conditions, characterized by “social disqualification” in Senegal: previous incarceration for nearly 50% of them and insufficient income to have autonomous housing (although homelessness is very uncommon in Senegal, unlike other African countries [21]).

The HIV prevalence found among heroin and/or cocaine users is seven times higher than among the general population and is strongly linked to the practice of injection (HIV prevalence of 9.4% in injectors) and sex (HIV prevalence of 13% in women). Women are also more exposed to HIV through injection (21.1% versus 7.5% for men) [24]. The HIV prevalence in DUs who had never used injection is four times higher than in the overall Senegalese population [5]. This might be due to the exposure to sexual risk through paid sex, underuse of condoms and multiple sexual partnerships in a context of precariousness [12,14]. These findings deserve further research to better understand the dynamics of the HIV epidemics in non-injecting DUs. Regarding the prevalence of HIV among females, the estimate found in our study is close to that of female sex workers in Senegal, shedding light on the necessity of taking into account drug use in prevention programmes among female sex workers in Africa, as already stated by others in West Africa [25].

With regard to hepatitis C, the rate found among DUs is more than 40 times higher than among the general Senegalese population (23.3% versus 0.49%) [15] and strongly linked to the practice of injection. The HCV prevalence of 38.8% in PWID is in line with that of the only study addressing the same issue and conducted in Ghana (40.1%) [9]. The HCV prevalence of 18% found in non-injecting DUs is also in line with other data [26], but it should be noted that this systematic review did not include data from sub-Saharan Africa. In developed countries, risk factors associated with HCV infection are age, injection [27] and more recently identified, crack consumption and unstable housing [17,28]. Those transmission routes that are also indicators of precariousness should therefore be explored in the context of developing countries and might partly explain the high rate of HCV infection in non-injectors.

Finally, the prevalence of HBs-Ag in the study was very similar to the prevalence of chronic hepatitis B in the Senegalese population. No risk factor associated with the use of drugs or to behavioural determinants has been identified. This is in line with the main route of transmission of HBV in sub-Saharan Africa being from mother to infant and during early childhood, therefore happening before the adult age.

The assessment of the level of knowledge among DUs regarding the determinants of HIV and HCV transmission was an essential component of this study. Most respondents had a good knowledge of HIV but not of HCV risk factors. A minority (13%) of DUs had shared needles in the past month, but 27 and 34% had shared water/filters and spoons, illustrating the fact that very few were aware of the risk of HCV transmission through shared injection equipment. HIV transmission through sexual intercourse was well known; however, only 50% used condoms. Commercial sex (at least once for drugs or money) was reported by a quarter of respondents, illustrating the obvious link with precariousness and high vulnerability in women. Only a very small number of participants had had an HIV, HBV or HCV serology performed prior to the study (29, 4 and 4%, respectively), although most of them knew that they were particularly at risk of being infected. These data question the accessibility to HIV and HCV screening for DUs populations in West Africa, in the absence of specific prevention and care programmes and in a repressive legal and social context for DUs. The significantly higher rate of participants (41.5%) who came for their results compared to those who were not included in the study (13.4%) shows the mobilizing character of participation in the survey. However, the most vulnerable, namely women, less than 34.1 years and PWID were very few, underlining the need for specific care programmes to reach them.

Some study limitations should be highlighted. First, the survey explored a precarious population and did not reach DUs from higher social classes where cocaine use seems to be more frequently reported. Second, despite having selected female and young “seeds,” the recruitment was very low among both groups. Our results may not be considered as fully representative of those two groups. Finally, we must take into account the methodological limitations related to the RDS approach. Indeed, although considered as the gold standard among methods for estimating the prevalence of infectious diseases among hidden populations, this method is relatively new and as such its robustness could not be fully compared to those based on random sampling. The study results should be considered rather as order of magnitude of the phenomenon and interpreted with caution.

More importantly, this study provided DUs with access to effective care and prevention through the field team and study staff. About 42% of participants came back to get their test results. Harm reduction activities were initiated during the study and continued thereafter through support from Expertise France Health Department, a bilateral French AIDS organization (www.expertisefrance.fr, last accessed 14 March 2015).

This study brings to light the significant vulnerability to HIV and HCV infection of non-injecting DUs living in developing countries. More studies are needed to better decipher the pathways to HCV infection in non-injecting DUs. Harm reduction programmes with access to substitution (including methadone) therapy are now required, and a response to the issues underlined by this study has been recently launched in Senegal: the DUs have been integrated as a vulnerable population in the National Strategic Plan in Response to AIDS (www.apf.francophonie.org/IMG/pdf/2013_10_vih_dakar_senegalstrategie.pdf, last accessed 14 February 2014) and fund raising has enabled the implementation of an integrated prevention and methadone programme for DUs in the Dakar area.
